# Loudness dependence of the auditory evoked potential: temporal stability, associations to sociodemographic variables, and functional significance—implications for clinical research

**DOI:** 10.3389/fnhum.2025.1507291

**Published:** 2025-02-06

**Authors:** Stein Andersson, Trine Waage Rygvold, Christoffer Hatlestad-Hall

**Affiliations:** ^1^Section for Clinical and Cognitive Neuroscience, Department of Psychology, University of Oslo, Oslo, Norway; ^2^Psychosomatic Medicine and CL Psychiatry, Division of Mental Health and Addiction, Oslo University Hospital, Oslo, Norway; ^3^Department of Clinical Neurosciences for Children, Oslo University Hospital, Oslo, Norway; ^4^Department of Neurology, Oslo University Hospital, Oslo, Norway

**Keywords:** LDAEP, test–retest reliability, sociodemographics, hormonal contraceptives, psychological distress, cognitive function

## Abstract

**Introduction:**

The loudness dependence of the auditory evoked potential (LDAEP) has been suggested as a biomarker for central serotonergic function, and as such a proxy for serotonin related psychiatric symptomatology and intervention outcome, particularly in depression. This study aims to explore LDAEP characteristics in a large healthy population by assessing its test–retest reliability and examining associations with sociodemographic variables, psychological distress, and performance-based cognitive function.

**Methods:**

Our sample included 100 healthy adults whose LDAEP was measured and correlated with age, sex, self-reported psychological distress, and cognitive performance.

**Results:**

Participants examined twice (*n* = 38) showed high test-retest reliability with intraclass correlations (ICCs) between 0.67 and 0.89 over a 2-to-3-month interval. Furthermore, the magnitude of the LDAEP was significantly higher in women than men, and female hormonal contraceptive users exhibited higher LDAEP than non-users. In females, age was inversely correlated with LDAEP. However, no significant associations were found between LDAEP and measures of psychological distress, including depressive symptoms, nor with cognitive test performance.

**Discussion:**

These results underline LDAEP's reliability as a biomarker over time, but also highlight age, sex and hormonal contraceptive use as significant factors influencing the LDAEP. Future research in clinical population should take these results into account, with an emphasis on providing the necessary sample sizes for relevant sub-group analyses.

## 1 Introduction

The loudness dependence of the auditory evoked potential (LDAEP), operationalized as the increase of the N1-P2 amplitudes of the auditory evoked potential (AEP) as a function of increasing stimuli intensity, was introduced in the early 1990s as a candidate electrophysiological biomarker of human central serotonergic function (Hegerl and Juckel, 1993). This hypothesis was further strengthened by animal research demonstrating an inverse relationship between the magnitude of the LDAEP response and serotonergic activity, as lower LDAEP was associated with higher serotonergic neuronal activity and vice versa (Juckel et al., 1997, 1999). These findings have since motivated research linking the LDAEP to various clinical conditions, especially in patients with affective disorders in which serotonergic activity and transmission are considered relevant for understanding key pathophysiological mechanisms (Moncrieff et al., 2023). However, studies examining associations between the strength of the LDAEP and psychiatric disorders such as major depression, bipolar disorders and schizophrenia and their symptomatology have yielded conflicting results (Park et al., 2010; Fitzgerald, 2024). More consistent evidence has suggested LDAEP as a predictor of treatment outcome in depressed patients administered selective serotonin reuptake inhibitors (SSRIs). Several studies have shown that depressed patients with pronounced LDAEP (indicative of low serotonergic activity) prior to treatment respond better to SSRIs compared to patients with weaker LDAEP (Hegerl et al., 2001; Lee et al., 2015), and a meta-analysis showed that stronger baseline LDAEP predicts favorable response to SSRIs (Yoon et al., 2021).

While numerous studies have explored LDAEP characteristics in clinical conditions, knowledge of LDAEP in healthy populations have mainly emerged from control samples matched to clinical groups, often with limited number of participants. Few studies have reported LDAEP characteristics from larger healthy populations in order to explore psychometric properties of the LDAEP and relationships between LDAEP and sociodemographic and clinical variables, knowledge that is crucial for the potential use of LDAEP as a biomarker in clinical research and intervention studies.

Test–retest reliability data has been reported in a few studies exploring LDAEP in healthy study populations. In an early dipole source activity analysis of the LDAEP, Hegerl et al. (1994) reported high test–retest stability with correlation coefficients above 0.80 of the tangential dipoles in 36 healthy participants over a 3-week time interval. Also Hensch et al. (2008) reported test–retest correlations of similar magnitude in 62 healthy participants tested 3 weeks apart. Although not being the main research question, Bamberg et al. (2021) explored the test–retest stability of LDAEP for 2 timepoints (~1 week apart) and reported high intra-individual stability in 37 young adult participants.

Several studies have reported LDAEP sex differences and association to age in both clinical and healthy control samples, mainly reporting higher LDAEP in females compared to males (Oliva et al., 2011; Jaworska et al., 2012) and reduced LDAEP with increasing age in healthy participants (Hegerl et al., 1994). However, few studies have examined how cognitive function is related to LDAEP. A recent study with 9 major depressed patients exploring LDAEP changes following electroconvulsive treatment (ECT) reported that pre-ECT LDAEP showed significant positive correlation with baseline cognitive function, as measured with the Repeatable Battery for the Assessment of Neuropsychological Status (RBANS) total score (Dib et al., 2024). In study of children with ADHD high LDAEP was associated with more inattention/impulsivity indexed as test score on a continuous performance task, and also correlated to the parents' rating of ADHD symptoms with more symptoms being associated with higher LDAEP (Park et al., 2022). Also, in healthy adults, higher levels of self-reported impulsivity have been associated with stronger LDAEP (Kim et al., 2016) which might reflect cognitive dysfunction related to aspects of executive functioning, a hypothesis that should be tested using performance-based cognitive assessment.

The aim of the present study is therefore two-fold: (1) to assess the test–retest reliability of LDAEP; and (2) to explore associations between demographic variables (age and sex), clinically meaningful variables, including self-reported psychological distress, and performance-based cognitive function, and LDAEP in healthy adults across the lifespan. Related to sex differences, exploratory analysis is performed in a subgroup of female participants to examine possible effect of using hormonal contraceptives (HC) as previous research has shown changes in the early components (N1 and P2) in auditory event-related potentials across the menstrual cycle, with the most prominent changes occurring during the luteal phase (Walpurger et al., 2004). Furthermore, the use of oral HC has been linked to an increased risk of developing a depressive episode and might affect serotonergic brain system and transmission (Larsen et al., 2020, 2022).

## 2 Material and methods

### 2.1 Participants

One hundred and eleven healthy subjects were recruited through local advertisement and social media. Before inclusion, participants were screened for previous or ongoing psychiatric or neurological disorders, substance abuse, and hearing impairment. Eleven participants were excluded because due to technical errors related to the EEG recording, leaving 100 participants (65 females, 35 males, mean age 37.6 years (±14.1) to complete the LDAEP protocol and subject to subsequent analyses. All participants provided informed written consent, and the study protocol was approved by the regional ethics committee for medical research (ref. no: 2016/2003).

Thirty-nine participants were randomly selected to complete a second LDAEP registration 2–3 months after their first visit. One participant displayed an extreme LDAEP difference (>3.5 SD from group mean) between the first and second LDAEP registration at all components, suggesting a technical error, and was consequently removed from further analyses, leaving 38 participants for analyzing test–retest reliability.

### 2.2 EEG registration and LDAEP paradigm

The LDAEP paradigm was part of a more extensive experimental protocol that also included a resting-state EEG recording and visual and auditory evoked potential paradigms with data and results presented elsewhere (Rygvold et al., 2021, 2022a,b; Hatlestad-Hall et al., 2022). The LDAEP paradigm was presented after a visual paradigm with no other auditory stimuli presented prior to the LDAEP paradigm onset, and the tones presented in the LDAEP paradigm did not overlap in intensity with tones presented in another auditory evoked potentials experiment conducted in the same session, to avoid possible interference effects. Participants were seated in a dimly lit, sound-attenuated room. Participants were screened with an audiograph to ensure normal hearing. Written instructions were provided on a 24” LCD screen (BenQ, model ID: XL2420-B) and participants were instructed to maintain their gaze on a red dot in the screen center throughout the presentation of the LDAEP stimuli. The LDAEP stimuli consisted of tones of 1,000 Hz presented at a duration of 50 ms and intensities of 55, 65, 75, 85, and 95 dB. Each intensity level was presented 80 times with a pseudo-randomized interstimulus interval varying between 1,200 and 1,800 ms, 5 ms edge ramp. Stimuli of equal intensity were never presented in two subsequent trials. Auditory stimuli were programmed in the Psychtoolbox-3 environment (Kleiner et al., 2007), run on a MATLAB platform (version 2015a; MathWorks, Natick, MA, USA) and generated by an Alto AMX-80 mixing console, and presented binaurally through Etymotic ER-1 insert earphones (Etymotic Research, Inc.).

EEG signals were obtained using a BioSemi ActiveTwo system (BioSemi B.V., Amsterdam), using a 64-channel BioSemi ActiveTwo headcap equipped with fixed AgAgCl electrodes placed in accordance with the international 10–20 system. Eye movements were recorded by four electrodes placed laterally and inferior/superior to each eye and additionally two external electrodes were attached to the earlobes, providing options for offline referencing. The sampling rate was 2,048 Hz, applying a 0.16 Hz high-pass filter to exclude low-frequency drifts. All EEG recordings were performed by author TWR.

Continuous EEG data were resampled to 512 Hz and re-referenced to the average of all EEG channels. EEG segments containing LDAEP data were extracted, excluding other EEG recordings not relevant to further processing of LDAEP data. A 1 Hz high-pass filter was applied to remove DC offset and low-frequency drifts. Channels with an amplitude SD outside an interval of 1–25 μV were removed from the reference signal iteratively. The ZapLine tool (de Cheveigné, 2020) and an upper bound 30 Hz low-pass filter were used to suppress line noise and high-frequency noise. Segments containing significant noise in more than 50% of the channels were rejected and remaining channels containing excessive noise in more than 10% of the data points were removed. Eye blinks and other movement artifacts were removed using independent component analysis (Delorme and Makeig, 2004). The automated independent component classifier ICLabel (Pion-Tonachini et al., 2019) was used to identify artifacts. The EEGLAB implementation of the second-order blind separation algorithm (Belouchrani et al., 1993) was used for component decomposition. A final removal of noisy channels was conducted with tools from the PREP pipeline toolbox (Bigdely-Shamlo et al., 2015). All the removed channels were then spherically interpolated. The continuous data were segmented into non-overlapping epochs, where each epoch consisted of a 100 ms pre-stimulus period and 450 ms post-stimulus period around each auditory stimulus. For baseline correction, the average signal between 100 ms pre-stimulus to stimulus onset was subtracted from the whole epoch. The epoch data were referenced to the linked mastoid signal (average of channels P9 and P10), and epochs with Fz, FCz or Cz amplitudes above 50 μV were rejected. The remaining epochs were averaged with respect to loudness level. The average number (SD) of included trials for each loudness level was: 71.5 (9.01) for 55 dB; 70.9 (9.58) for 65 dB; 70.9 (9.22) for 75 dB; 70.6 (10.70) for 85 dB; and 68.6 (11.18) for 95 dB.

The LDAEP was calculated from the N1, P2, and N1/P2 peak-to peak amplitudes at midline electrodes Fz, FCz, and Cz from the averaged auditory evoked potential waveforms for each participant. Time windows were predefined as the most negative amplitude value 60–140 ms for the N1, and the most positive amplitude value 150–250 ms for the P2 component. Manual selection of amplitude peaks was performed if their latencies deviated from these specified ranges. According to conventions, LDAEPs for the N1, P2, and N1/P2 peak-to-peak amplitudes are expressed as the slopes of the respective linear regression lines (μV/10 dB). Based on established practices in auditory evoked potential research (Picton et al., 1974), including earlier LDAEP research (Mulert et al., 2007; Linka et al., 2009), the fronto-central midline channels Fz, FCz and Cz were selected for statistical analyses.

### 2.3 Self-reported psychological distress

The participants completed two questionnaires expressing self-reported psychological distress: The Beck Depression Inventory (BDI-II) and the Perceived Stress Scale (PSS-10). The BDI-II (Beck et al., 1996) is a validated 21 item questionnaire, where items are scored 0–3), reporting depressive symptoms during the last 2 weeks. The BDI-II is widely used in clinical studies to quantify the severity of depressive symptoms or as an outcome measure in clinical trials. In healthy subjects, a cut-off point of BDI-II total score ≥10 is suggested to identify individuals with some degree of depressive symptoms closely related to dysphoria or clinically relevant mild depression (Kendall et al., 1987; Koster et al., 2005). The PSS-10 is a questionnaire intended to measure the degree of perceived stress relative to the ability to cope with stressful situations experienced during the last month (Cohen et al., 1994; Taylor, 2015). Although not designed for diagnostic purpose, the PSS-10 have been proven useful in identifying prodromal stages of psychiatric disorders (Cohen et al., 1994).

### 2.4 Cognitive assessment

Participants completed a neuropsychological test battery covering several cognitive domains: Attention/working memory [Digit span forward, Digit span backward and number sequencing from the Wechsler Adult Intelligence Scale IV (WAIS-IV) (Wechsler, 2008)] and total accuracy score from the Ruff 2&7 selective attention test (Ruff et al., 1992); executive function [Trail Making Test condition 4, Color-Word Interference Test conditions 3 and 4, and the Verbal fluency category switching from the Delis-Kaplan Executive Function System (D-KEFS) (Delis et al., 2001); verbal memory (Rey Auditory Verbal Learning Test (Schmidt, 1996))] and visual memory [the Aggie Figures Learning Test (Majdan et al., 1996)]. Raw scores were transformed to *T-*scores using relevant normative data from the corresponding test manuals. Domain *T-*scores represent the mean of individual test *T-*scores within the respective domains. A global cognitive index score was calculated as the mean of cognitive domain *T-*scores.

### 2.5 Statistical analyses

To analyze associations between LDEAP and categorical variables, linear mixed model analyses with subsequent *post hoc* pairwise tests were implemented in R using packages “lme4” and “emmeans”. Degrees of freedom were estimated with the Kenward-Roger approximation method, as implemented in the R package “pbkrtest” (Halekoh and Højsgaard, 2014). Associations between LDAEP and continuous variables (age, self-reported psychological distress, and cognitive performance) were analyzed using bivariate Pearson's correlation coefficient. To control for Type I error rate when conducting multiple statistical tests, Bonferroni correction was employed in correlation analyses to adjust for multiple comparisons by dividing the alpha level (0.05) with number of tests included in the analyses. Temporal stability, or test–retest reliability, was reported as bivariate Pearson's correlation coefficients and intraclass correlation coefficients (ICC) using a two-ways mixed model with absolute agreement, reported as average measures [ICC(3,*k*)]. These ICC parameters account for the origin of error or random effect (Shrout and Fleiss, 1979), and the absolute agreement ICC measures to what degree the actual values are similar, although they might be linearly related, thereby representing a more conservative estimate of reliability compared to the Pearson correlations (McGraw and Wong, 1996). Absolute agreement thus represents a more conservative estimation of reliability than Pearson correlations. Common classification guidelines for level of ICC state below 0.50 as poor; 0.50–0.75 as moderate; 0.75–0.90 as good; >0.90 as excellent (Koo and Li, 2016), although others propose less conservative criteria stating ICC 0.60–0.75 as good and >0.75 as excellent (Cicchetti, 1994). Supplementary analyses exploring differences in BDI-II, PSS-10 and cognitive domain scores between females and males and between females using and not using HC were performed with student's-tests with effect sizes reported as Cohen's *d*.

## 3 Results

Sociodemographic characteristics (age, sex, education) of the participants, the scores on the BDI-II and PSS-10 measuring psychological distress, and the neuropsychological test performance measuring cognitive function are presented in Table 1.

**Table 1:** Subject characteristics (*N* = 100).

	**Mean (±SD)**	**Min.–Max**.
**Demographics**		
Age (years)	37.6 (±14.1)	18–71
Gender (m/f)	35/65	
Women <42 years using/not using hormonal contraceptives (n)	19/24	
Education (years)	14.6 (±2.21)	9–18
**Psychological distress**		
Beck depression inventory-II (total score)	4.83 (±5.59)	0–26
Perceived stress scale (total score)	10.80 (±5.46)	0–27
**Cognitive function (domain** ***T-*****scores)**		
Attention/working memory	51.0 (±6.93)	33.3–68.6
Executive function	54.9 (±7.84)	28.3–66.7
Processing speed	53.8 (±5.75)	34.5–64.7
Visual memory	50.8 (±9.44)	22.7–63.8
Verbal memory	59.5 (±8.37)	32.0–73.7
Global cognitive index	54.3 (±5.55)	36.7–63.0

**Figure 1:**
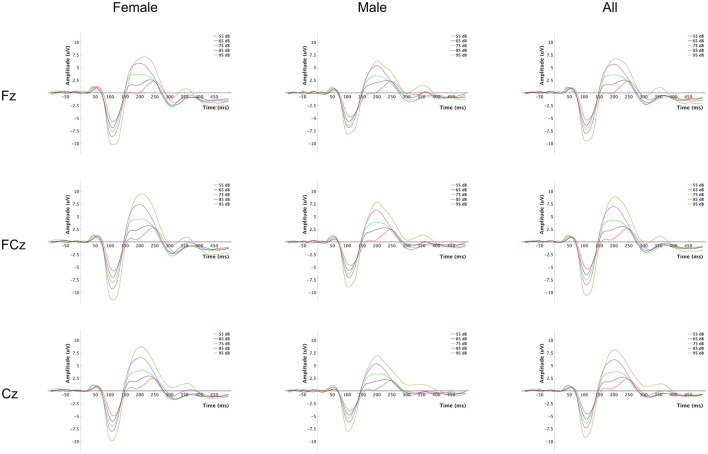
Event-related potential (ERP) waveforms for each tone intensity level. The plot grid columns represent each group (females, males, and all), and the rows represent the EEG channels at which the signals were recorded.

**Figure 2:**
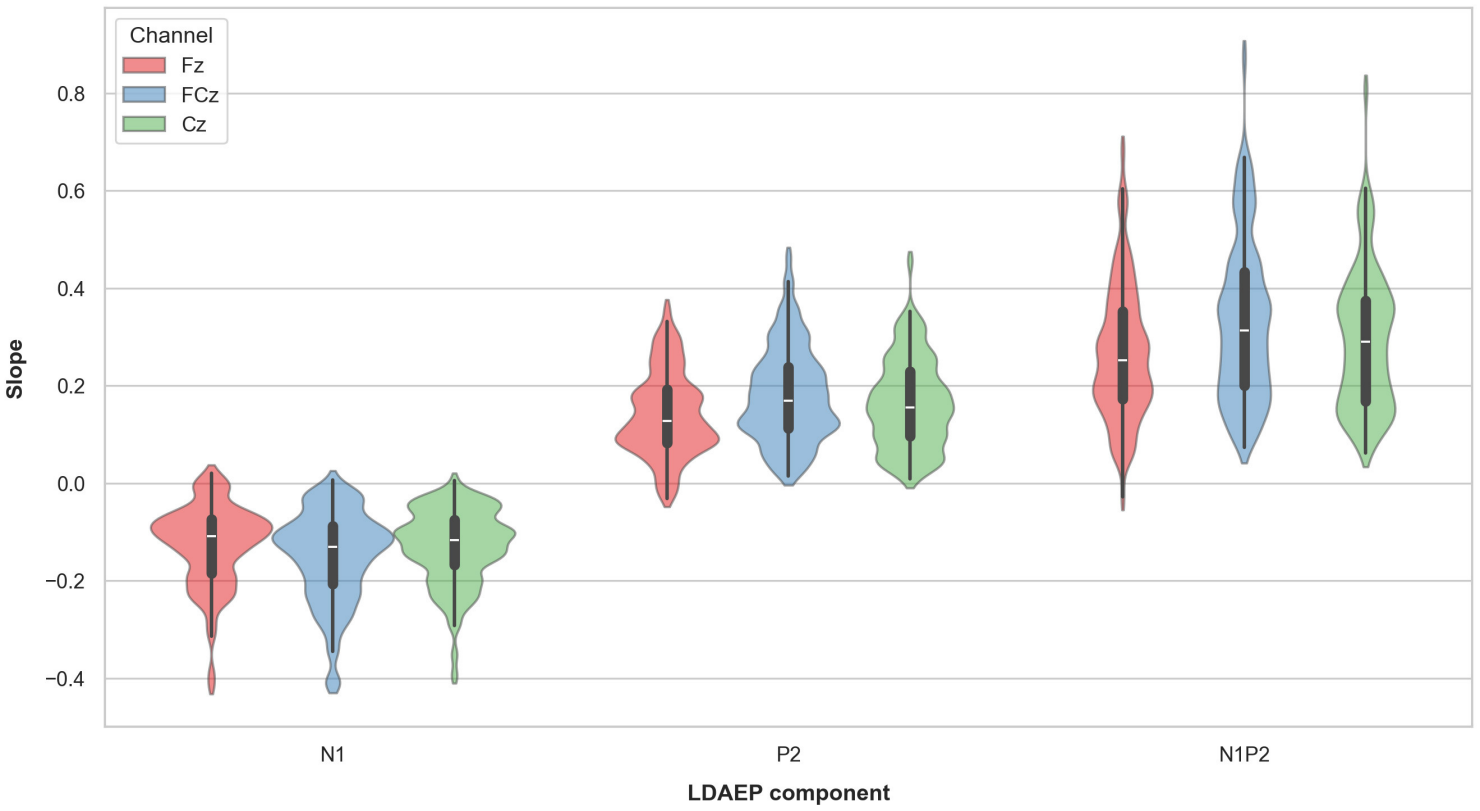
Violin plots representing the LDAEP (N1, P2, and the N1-P2 peak-to-peak measure) by channel.

The event-related potential (ERP) waveforms at Fz, FCz and Cz of the LDAEP paradigm for each tone intensity level are presented in Figure 1, for all participants and separately for females and males. A complete overview of mean amplitude values for N1, P2, and N1P2 peak-to-peak components at Fz, FCz and Cz across the 5 loudness intensities and the LDAEP slope values (μV/10 dB) are reported in Table 2. The latter is also presented in Figure 2. For further statistical analyses, only the LDAEP values of the N1P2 peak-to-peak amplitude for electrodes Fz, FCz, and Cz are reported.

**Table 2:** Descriptive statistics (*N* = 100) of mean amplitudes, LDAEP defined as the slope of the linear regression line (μV/dB) and mean test–retest difference (*N* = 38) for all components measured from midline electrodes Fz, FCz, and Cz.

	**Mean amplitudes (μV) 55/65/75/85/95 dB**	**LDAEP (μV/10 dB) Mean (SD)**	**Min–Max**	**Test–retest LDAEP mean difference (SD)**
**Fz**				
N1	−5.98/−7.06/−7.94/−8.97/−11.33	−0.126 (0.083)	−0.42 to 0.02	0.002 (0.060)
P2	3.21/4.07/5.13/7.17/8.68	0.140 (0.086)	−0.03 to 0.36	0.011 (0.074)
N1P2	9.20/11.12/13.07/16.14/20.01	0.267 (0.137)	−0.03 to 0.68	0.012 (0.077)
**FCz**				
N1	−5.99/−7.15/−8.18/−9.47/−12.34	−0.150 (0.093)	−0.41 to 0.41	0.012 (0.059)
P2	3.66/4.64/5.82/8.47/10.76	0.180 (0.097)	0.02–0.46	0.012 (0.071)
N1P2	9.64/11.79/14.00/17.94/23.10	0.331 (0.164)	0.07–0.87	0.024 (0.079)
**Cz**				
N1	−5.10/−6.089/−7.03/−8.14/−10.58	−0.130 (0.077)	−0.40 to 0.01	0.008 (0.058)
P2	3.28/4.12/5.27/7.55/9.72	0.163 (0.092)	0.01–0.46	0.007 (0.062)
N1P2	8.38/10.20/12.30/15.69/20.29	0.293 (0.144)	0.06–0.81	0.020 (0.080)

### 3.1 Temporal stability

**Figure 3:**
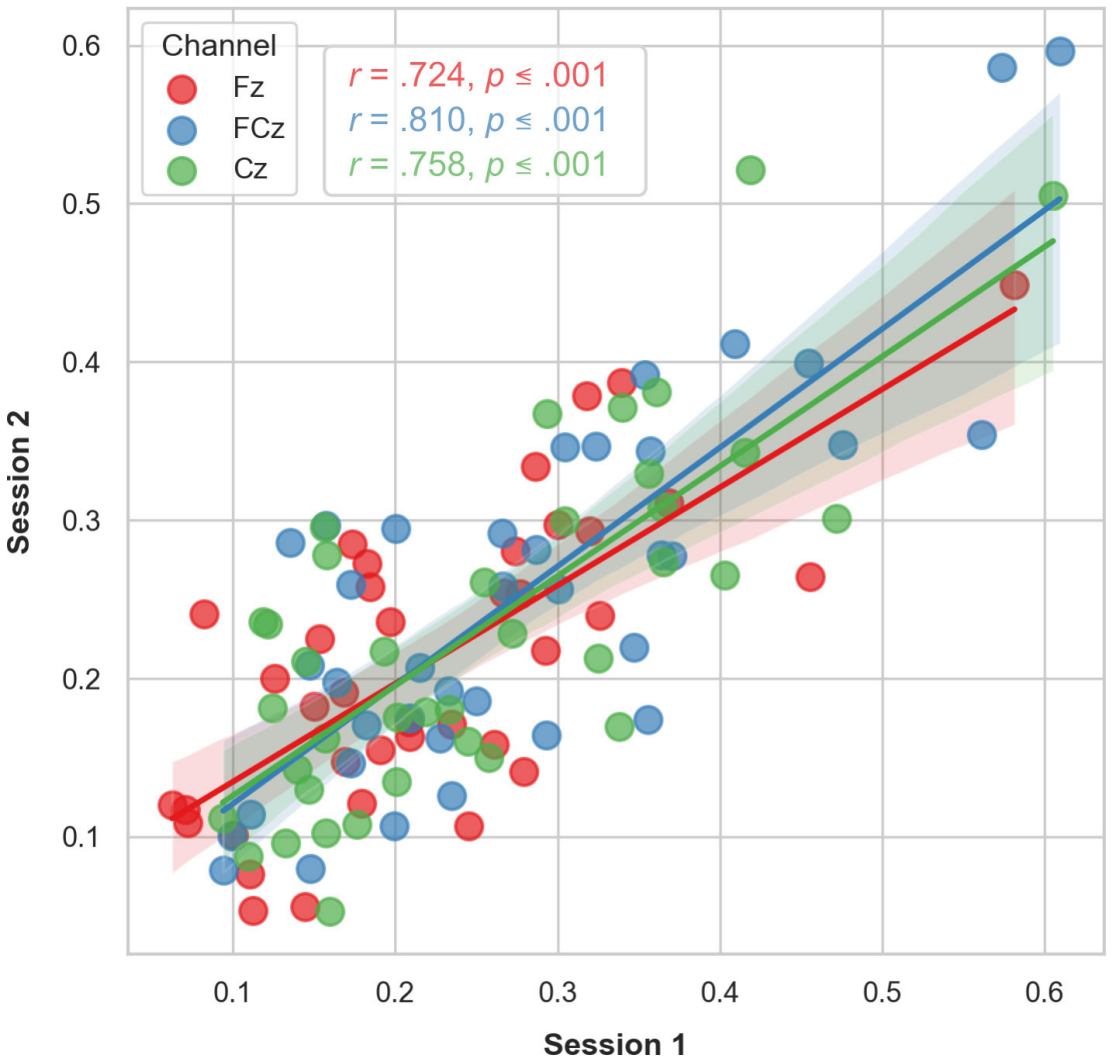
Scatter plot of the LDAEP observed at each timepoint for the subset of participants (*n* = 38) who underwent two longitudinal LDAEP recording sessions. The regression lines indicate the observed correlations between the two timepoints. The *shaded area* represents the 95% confidence interval of the regression lines.

Mean differences and standard deviations of the LDAEP slopes of N1, P2, and N1P2 are presented in Table 2. Correlation analyses for the LDAEP slope of the N1P2 at Fz, FCz, and Cz between timepoints were all significant (*p* ≤ 0.001) with correlation coefficients of 0.724, 0.810, and 0.758, respectively (Figure 3). Intraclass correlation (ICC) analysis of the N1P2 slope yielded ICC coefficients in the moderate and good range according to Koo and Li (2016) classification with ICC = 0.834 (95 % CI = 0.683–0.914) for Fz, ICC = 0.887 (95 % CI = 0.781–0.942) for FCz and ICC = 0.856 (95 % CI = 0.724–0.925) for Cz.

### 3.2 Sex differences and associations with age

**Figure 4:**
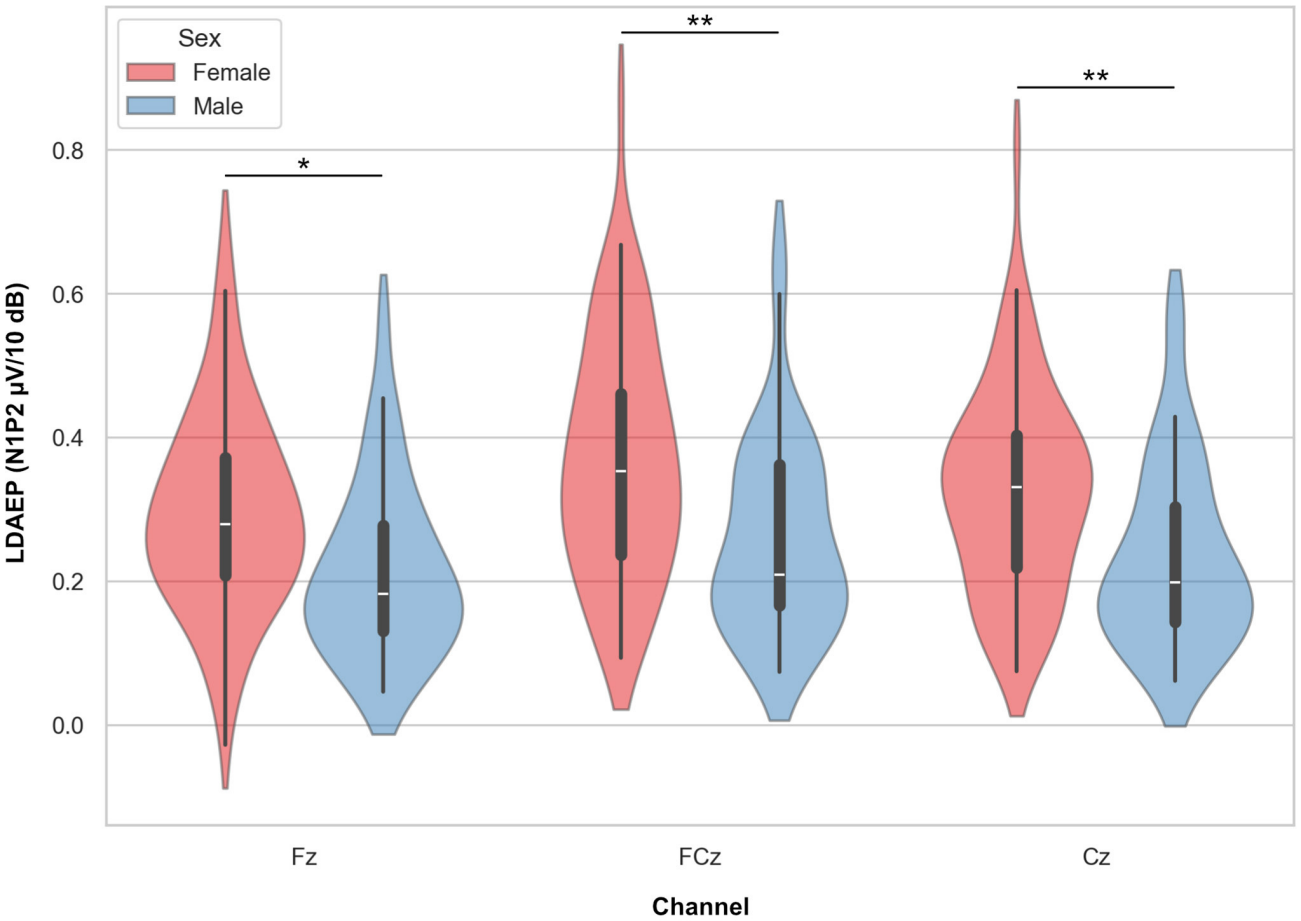
Violin plots representing the LDAEP by sex. *Asterisks* indicate significant differences between groups, as indicated by *post hoc* testing, for each channel separately (* = *p* < 0.05, ** = *p* < 0.01).

A mixed linear model with N1P2 slope as the dependent variable, channel (Fz, FCz, and Cz) as a within-subject factor, sex as a group factor, and age as a covariate, showed a significant effect of sex, with females showing higher slope values (β = −0.084, SE = 0.029, *p* = 0.004, 95% CI = [0.141, −0.027]). There were no significant main effects of channel or interaction effect of channel by sex, but a significant effect of age (β = −0.003, SE = 0.001, *p* < 0.001, 95% CI = [−0.004, −0.001]) showing a reduction in LDAEP slope with increasing age (see below). *Post hoc t*-tests with marginal means adjustments showed significantly higher slope values for all channels in females compared to males with medium Cohen's d effect sizes ranging from 0.62 to 0.69 (Table 3A; Figure 4).

**Table 3:** (A) Sex differences for the LDAEP defined as the N1P2 slope (μV/10 dB) value, (B) LDAEP differences between females under age 42 using or not using hormonal contraceptives (HC).

**(A) LDAEP**	**Females (*n =* 65) mean/EMmean^a^ (SE)^b^**	**Males (*n =* 35) mean/Emmean^a^ (SE)^b^**	***t***	***p***	***d* ^c^**
Fz	0.295/0.293 (0.017)	0.214/0.218 (0.023)	2.592	0.011	0.62
FCz	0.369/0.366 (0.017)	0.260/0.264 (0.023)	3.513	0.001	0.69
Cz	0.325/0.329 (0.017)	0.235/0.239 (0.023)	2.889	0.005	0.65
**(B) LDAEP**	**Females**<**42 years using HC (*****N** =* **19) mean/EMmean**^a^ **(SE)**^2^	**Females**<**42 years not using HC (*****N*** = **24) mean/EMmean**^a^ **(SE)**^b^	***t***	***p***	***d** ^c^*
Fz	0.363/0.347 (0.033)	0.277/0.274 (0.021)	1.810	0.074	0.58
FCz	0.479/0.464 (0.033)	0.344/0.329 (0.021)	3.313	0.001	0.81
Cz	0.411/0.396 (0.033)	0.303/0.295 (0.021)	2.483	0.015	0.79

a Estimated marginal mean;

b Standard error;

c Cohen's d effect size.

**Figure 5:**
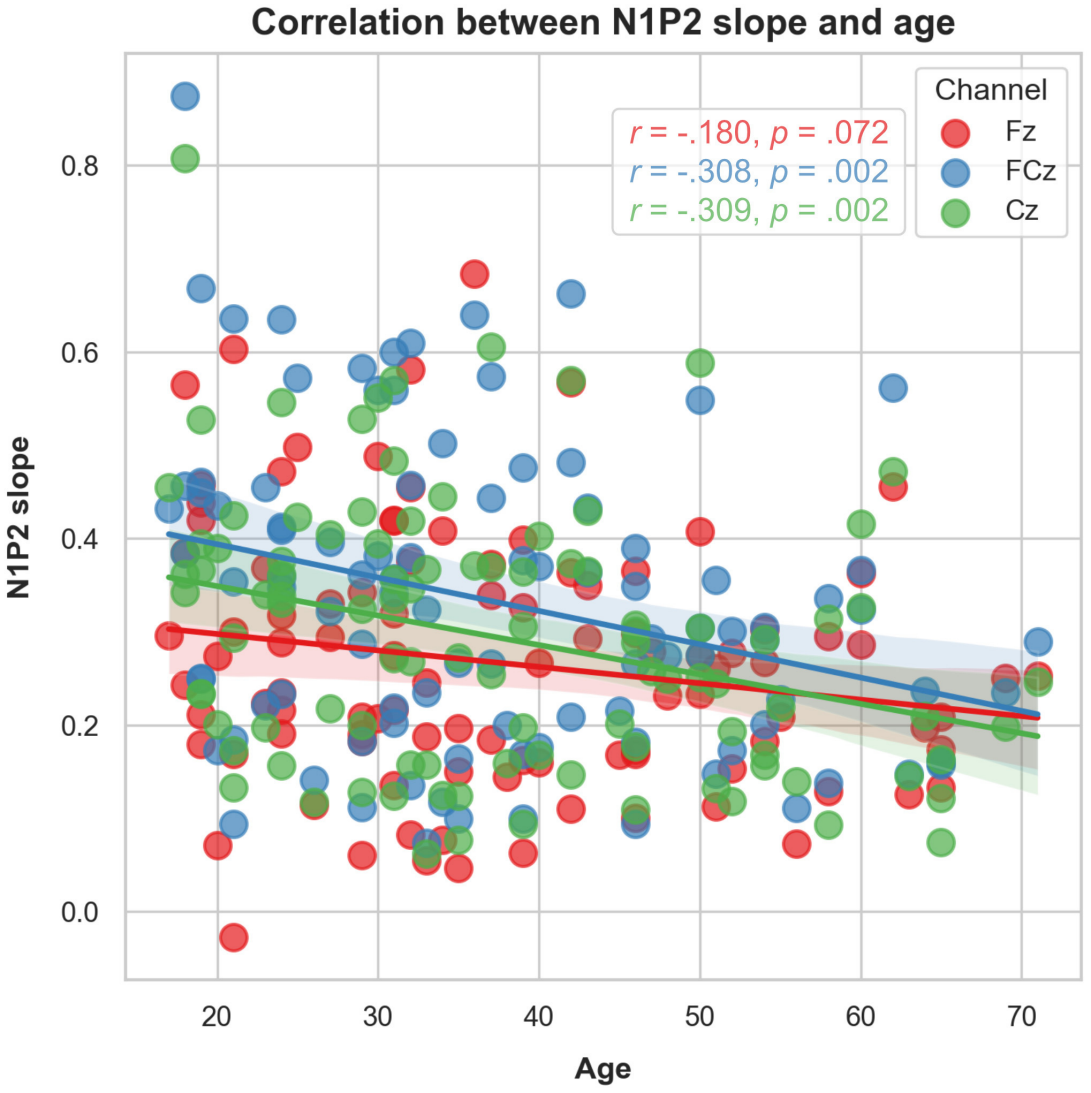
Scatter plot representing the association between age and the LDAEP by channel.

**Figure 6:**
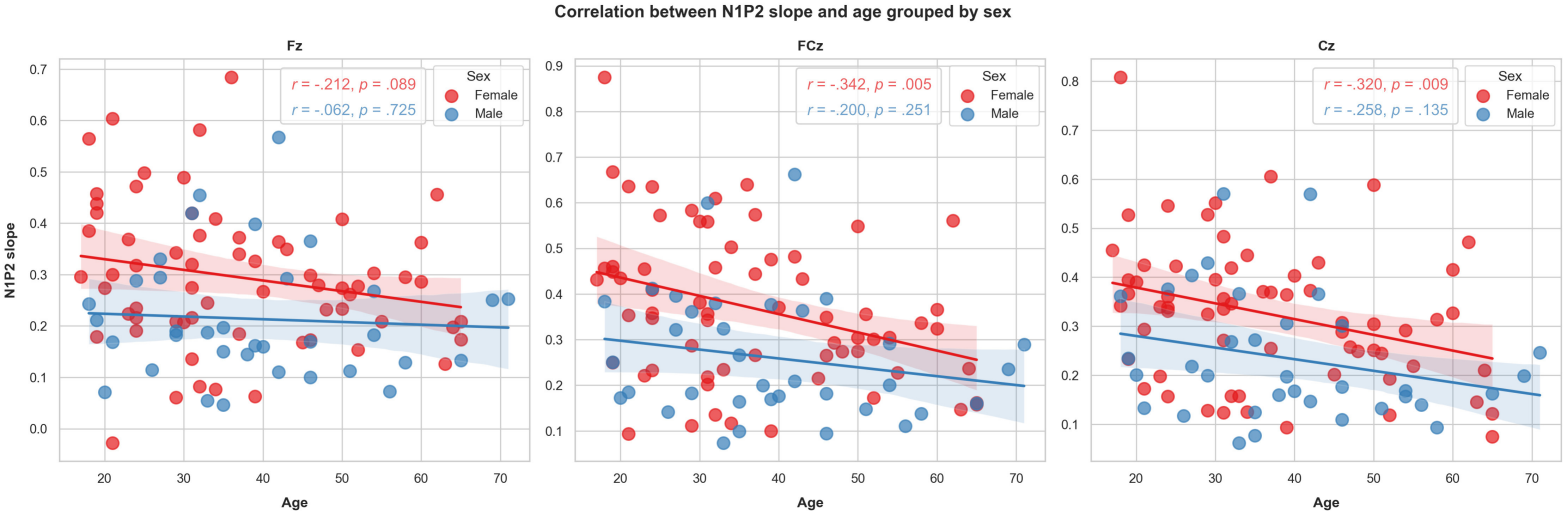
Scatter plots representing the association between age and the LDAEP by sex and channel.

Increasing age was significantly associated with lower N1P2 slope values for FCz (*r* = −0.308, *p* = 0.002) and Cz (*r* = –0.309, *p* = 0.002). The correlation was not significant for Fz (*r* = –0.180, *p* = 0.072). Adjusting *p-*value level for multiple comparisons by Bonferroni's correction (0.05/4), the FCz and Cz correlations remained significant (*p* < 0.017). See Figure 5. Analysing the N1P2 slope and age associations for males (*n* = 35) and females (*n* = 65) separately (Figure 6) yielded significant correlations for women at FCz (*r* = –0.342, *p* = 0.005) and Cz (*r* = –0.320, *p* = 0.009), but not at Fz (*r* = –0.212, *p* = 0.089). For males there were no significant associations between age and N1P2 slope.

### 3.3 Effects of hormonal contraceptives

**Figure 7:**
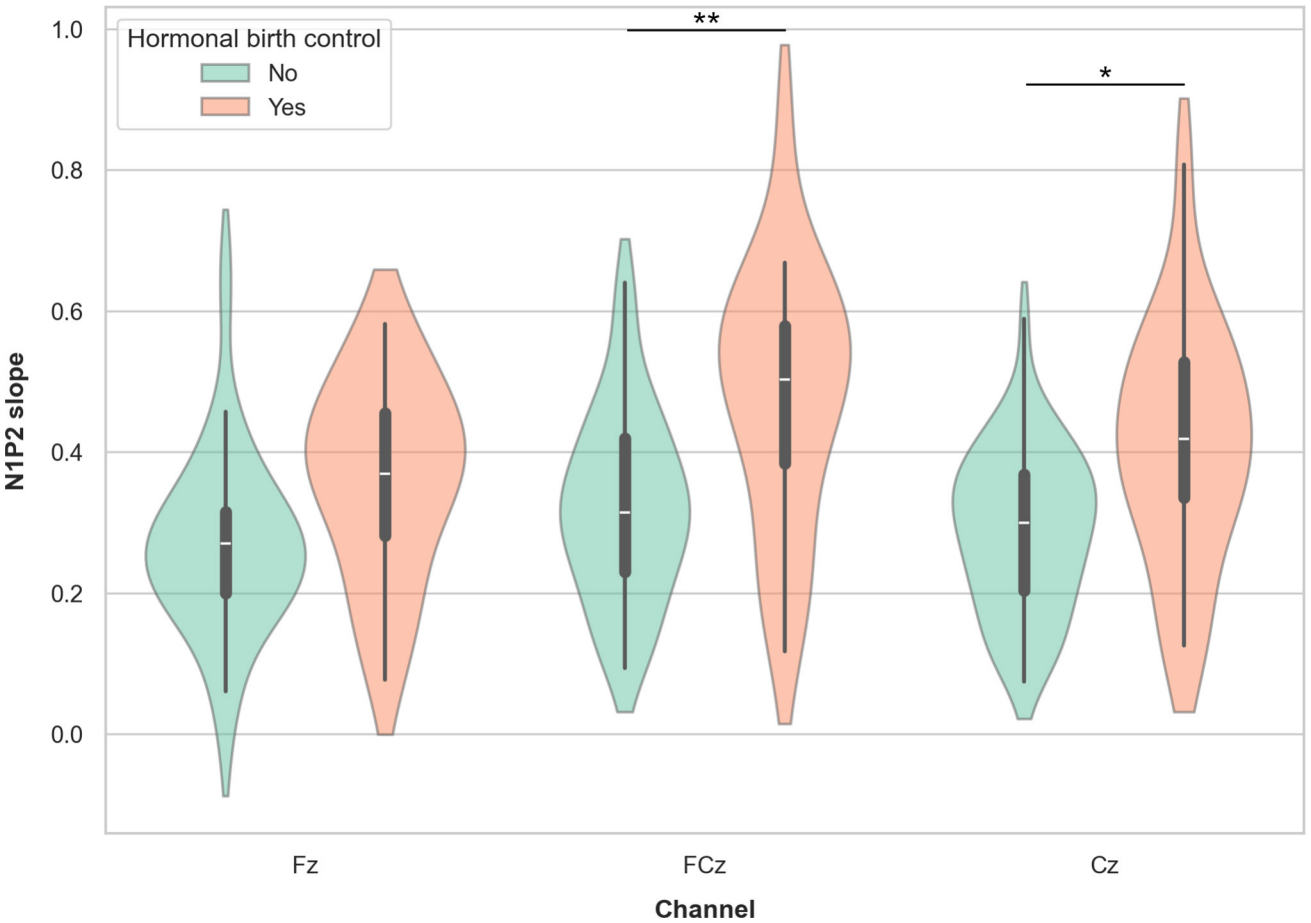
Violin plots representing the LDAEP in females currently using and not using hormonal contraceptives. *Asterisks* indicate significant differences between groups, as indicated by *post hoc* testing, for each channel separately (* = *p* < 0.05, ** = *p* <0.01).

Among females, 19 participants in the age range 18–42 years reported using hormonal contraceptives (HC), while 24 reported not using HC. A supplementary mixed linear model with N1P2 slope as the dependent variable, channel (Fz, FCz, and Cz) as a within-subject factor, HC use (yes/no) as a group factor, and age as a covariate showed a significant effect of HC use (β = 0.101, SE = 0.038, *p* = 0.009, 95% CI = [0.025, −0.176]) with females using HC showing higher LDAEP slope values. There was no main effect of channel, nor significant interaction effect of channel by HC use. There was a small but significant effect of age (β = −0.002, SE = 0.001, *p* = 0.021, 95% CI = [−0.003, −0.000]) showing a reduction in LDAEP slope with increasing age. *Post hoc t-tests* with marginal means adjustments showed significantly higher slope values FCz and Cz in females using HC compared non-users with medium to strong Cohen's d effect sizes ranging from 0.58 to 0.81 (Table 3B; Figure 7).

### 3.4 Associations with psychological distress

No significant associations between self-reported depressive symptoms measured (total BDI-II score) and the N1P2 slope for Fz, FCz, and Cz, with correlation coefficients ranging from −0.066 to −0.105. Using a cut-off point of ≥10 to group individuals with or without subclinical depression identified that 15% of participants had subclinical depressive symptoms. There was no significant N1P2 slope difference between groups when using this cuf-off to dichotomize subject with and with-out sub-clinical depressive symptoms. Correcting for age in a partial correlation analysis did not reveal any significant associations between N1P2 slope values and self-reported symptoms of depression, nor did running correlation analyses for males and females separately. Similarly, no significant correlations were observed between total PSS score and any N1P2 slope value (*r* between 0.075 and 0.085). As shown in Supplementary Table S1, there were no significant differences in BDI-II or PSS-10 scores between males and females. However, females using HC displayed a significant lower BDI-II score (*p* = 0.032) compared to females not using HC.

### 3.5 Cognitive function

There were no significant correlations between neuropsychological performance score on any cognitive domain, including the global cognitive index, and N1P2 slope values at any electrode with correlation coefficients ranging from *r* = –0.071 to 0.146). Female participants performed significantly better than males on verbal memory (*p* < 0.001), visual memory (*p* = 0.015) and global cognitive index score (*p* = 0.005). Consequently, supplementary correlation analyses were performed for male and female participants separately, still showing no significant association between LDAEP and cognitive performance. Female participants not using HC performed significantly better compared to HC users on several cognitive domains, including processing speed (*p* = 0.019), visual memory (*p* = 0.002) and global cognitive index (*p* = 0.004). Supplementary analyses for HC users and non-users separately showed no significant correlations between LDAEP and cognitive performance when controlling for multiple comparisons.

## 4 Discussion

The main results of this study of LDAEP characteristics in a healthy population are that LDAEP measures display satisfactory to high temporal stability over a 2–3 month test–retest interval, with intraclass correlations (ICC) between 0.67 and 0.89. To our knowledge, measures of long interval test–retest reliability has not been previously reported in a large sample of healthy subjects. We argue for the importance of this finding as it implies that changes in LDAEP response over time may be considered a reliable index of altered neurophysiological processes. This is crucial for the validity of studies using LDAEP in repeated measures designs, including clinical trials investigating intervention effects. Our results are in line with previous studies, e.g., Hensch et al. (2008) who reported comparable test–retest reliability results over 3 weeks in a student population, Beauducel et al. (2000) who reported high temporal stability in a 2–4 weeks interval, and Bamberg et al. (2021) who reported good test–retest reliability over a 1-week interval. However, our study employed a test–retest time interval of 3 months which is closer to common follow-up timepoints in clinical trials.

Sensory evoked potentials vary over the adult age span, with a generally observed amplitude decrease and latency increase of early and late evoked potential components from adult to older age (Onofrj et al., 2001). In our study, the magnitude of the LDAEP was significantly correlated with age with higher LDAEP values being associated with decreasing age. However, this was true for females only, a difference that could be explained by less statistical power, considering that there were nearly twice as many female participants than male participants. Interestingly, few studies have explicitly reported age effects in larger healthy populations, although LDAEP and age associations are reported in clinical and smaller healthy control samples. Similarly to our results, Hegerl et al. (1994) reported a significant negative correlation between LDAEP and age in 40 healthy subjects. However, Jang et al. (2022) reported significant positive LDAEP-age correlations in patient groups with major depression and schizophrenia, but not in a control group of 35 healthy participants. Similarly, Ostermann et al. (2012) reported significant LDAEP-age correlations in patients with affective disorders, but no age effects in 40 healthy controls.

Among the participants, women displayed significantly higher LDAEP values compared to men. This finding is in line with previous research on LDAEP sex differences in both healthy individuals (Oliva et al., 2011) and in clinical samples of patients with major depressive disorder (Jaworska et al., 2012), reporting steeper LDAEP in females relative to men. This sex difference may be viewed in connection with previous studies reporting lower serotonergic transmission in females compared to men (Nishizawa et al., 1997) which could be further linked to the observed sex differences in the prevalence of affective disorders (Van de Velde et al., 2010).

In exploratory sub-analyses we found that women using hormonal contraceptives exhibit significantly larger LDAEPs compared to age-matched women not using hormonal contraceptives. Although debated, it has been suggested that depression might represent a potential adverse effect of hormonal contraceptive use (Schaffir et al., 2016; Skovlund et al., 2016) and interfere with emotional processing mechanisms (Lewis et al., 2019). The increased risk of depression in hormonal contraceptive users might be related to lowered levels of serotonin receptor binding found in healthy oral contraceptive users compared to non-users (Larsen et al., 2020). This aligns well with our current findings that increased LDAEP is associated with hormonal contraceptive use. However, these findings need replication in further studies employing designs specifically developed to investigate the relationship between LDAEP and use of hormonal contraceptives.

We observed no significant associations between LDAEP and self-reported measures of psychological distress. Although LDAEP has been suggested to predict SSRI treatment response in major depression (Yoon et al., 2021), studies of LDAEP association to diagnostic criteria or self-reported affective symptoms in clinical samples have shown conflicting results (Fitzgerald, 2024). Few studies have reported on the associations between LDAEP and severity of self-reported depressive symptoms and other psychological variables in non-clinical samples. In 157 healthy individuals, Kim et al. (2016) found that participants with high LDAEP showed significantly increased impulsivity scores compared to those with low LDAEP. High LDAEP was also significantly associated with more self-reported symptoms of depression. In the current study we used the BDI-II to assess the level of depressive symptoms, and typically BDI-II scores in non-clinical samples will be skewed toward very low scores. Nevertheless, applying a cut-off BDI-II score of ≥10, we did not observe any difference in LDAEP between sub-clinically depressed and non-depressed. In our study females using hormonal contraceptives reported lower BDI-II scores compared to HC non-users. Although based on small sizes and must be interpreted with caution, this finding is surprising as HC users show stronger LDAEP than non-users which could indicate more depressive symptoms. As for other findings in this study addressing the impact of HC use these results need confirmation in larger studies designed to explore HC use explicitly.

To our knowledge, no previous studies have reported on associations between LDAEP and performance-based cognitive function in adults. One study in children with ADHD found no significant association between IQ score and LDAEP (Park et al., 2022). They did, however, find that LDAEP was positively correlated with questionnaire-rated level of inattention and hyperactivity-impulsivity. Also, in adults, higher levels of self-reported impulsivity have been associated with stronger LDAEP, indicating lower serotonergic activity (Kim et al., 2016). Therefore, it could be hypothesized that performance-based measures of aspects of executive functioning capturing impulsivity and lack of inhibitory control, would be associated with stronger LDAEP. However, this was not evident in our sample of healthy participants. Future studies should address this in clinical samples.

### 4.1 Strengths and limitations

Compared to most studies employing a LDAEP paradigm, this study has a relatively large number of participators across the adult lifespan (18–71 years). However, a majority were females, making the analyses within the male subgroup less powerful. Also, the results concerning contraceptive users among the females is based on a small sample size and needs to be replicated in studies designed to address these particular research questions. Several state dependent factors, e.g., caffeine, nicotine or nutritional intake that might influence auditory evoked potentials and LDAEP, were not controlled for in this study. As summarized by O'Neill et al. (2008), studies employing tryptophan depletion aimed to induce a reversible reduction of central serotonergic function have shown no convincing evidence suggesting that LDAEP is sensitive to acute changes in serotonergic neurotransmission, questioning its utility as a marker of central serotonergic function. However, more recent research examining the effect of fasting vs. carbohydrates or protein consumption on LDAEP, reported significant nutrition-specific effect on LDAEP and blood-glucose levels with LDAEP being lower during satiety, irrespective of the type of food (Bamberg et al., 2021).

## 5 Conclusions and implication for clinical research

This study demonstrates that LDAEP show acceptable to high temporal stability over a two to three-months period, making it a reliable index of change and thus suitable for intervention studies requiring repeated measures of LDAEP. However, future studies using LDAEP paradigms to characterize clinical populations and using LDAEP as a predictor for treatment outcome or response to intervention should provide large enough sample sizes to control for sex differences and age-related changes, and to provide sufficient statistical power to perform subgroup analyses. Furthermore, special consideration should be given to samples of younger female participants, as the use of hormonal contraceptives may interact with serotonergic transmission, which in turn could affect the magnitude of the LDAEP.

## Data Availability

The raw data supporting the conclusions of this article will be made available by the authors, without undue reservation.
