# Supplementary material for: Loudness dependence of the auditory evoked potential: temporal stability, associations to sociodemographic variables, and functional significance—implications for clinical research
Source: Front Hum Neurosci. 2025 Feb 6;19:1507291. doi: 10.3389/fnhum.2025.1507291 (PMC11839817; doi:10.3389/fnhum.2025.1507291)
Supplement: Supplementary file 1 [file Data_Sheet_1.pdf]

Supplementary table 1.

A: Sex differences for the Beck Depression Inventory (BDI-II) total score and Perceived Stress Scale (PSS) total score.

B: Beck Depression Inventory (BDI-II) total score and Perceived Stress Scale (PSS) total score differences between females under age 42 using or not using hormonal contraceptives (HC).

**A**	**Females (n = 65)**<br>**Mean (SD)**	**Males (n = 35)**<br>**Mean (SD)**	**t**	**p**	**d**[3]
BDI-II[1] total score	4.27 (5.262)	5.262 (5.987)	-1.050	0.296	-0.22
PSS[2] total score	11.56 (5.345)	9.69 (4.733)	1.737	0.086	0.37
**B**	**Females <42 years**<br>**using HC (N = 19)**<br>**Mean (SE)**	**Females <42 years**<br>**not using HC (N=24)**<br>**Mean (SE)**	**t**	**p**	**d**[3]
BDI-II[1] total score	2.53 (2.366)	6.30 (7.087)	-2.220	0.032	-0.69
PSS[2] total score	11.79 (5.553)	13.46 (5.397)	-0.994	0.326	-0.31

[1]Beck Depression Inventory-II; [2]Perceived Stress Scale; [3]Cohen’s d

Supplementary table 2.

A: Sex differences in neuropsychological performance score for different cognitive domains.

B: Neuropsychological performance score (cognitive domains) differences between females under age 42 using or not using hormonal contraceptives (HC).

**A** **Cognitive domain (T-score)**	**Females (n = 65)** **Mean (SD)**	**Males (n = 35)** **Mean (SD)**	**t**	**p**	**d[1]**
Attention/Working memory	51.25 (6.838)	50.46 (7.093)	0.525	0.601	0.11
Executive function	56.21 (5.791)	54.22 (8.773)	1.324	0.189	0.29
Processing speed	54.27 (5.109)	54.14 (6.481)	.107	.915	0.02
Verbal memory	61.86 (5.692)	54.72 (9.081)	4.774	<.001	1.01
Visual memory	51.95 (8.688)	47.35 (9.254)	2.470	.015	0.52
Global cognitive index	55.13 (4.523)	51.93 (6.498)	2.886	.005	0.61

**B** **Cognitive domain (T-score)**	**Females <42 years using HC (N = 19)** **Mean (SD)**	**Females <42 years not using HC (N = 24)** **Mean (SD)**	**t**	**p**	**d[1]**
Attention/Working memory	49.54 (6.697)	53.13 (5.668)	-1.834	0.075	-0.59
Executive function	54.54 (7.165)	57.12 (4.815)	-1.396	0.170	-0.44
Processing speed	52.32 (5.550)	55.90 (3.648)	-2.451	0.019	-0.78
Verbal memory	59.64 (6.585)	62.39 (4.127)	-1.661	0.105	-0.52
Visual memory	51.02 (9.182)	57.71 (3.162)	-3.336	0.002	-1.03
Global cognitive index	53.42 (5.454)	57.28 (2.724)	-3.029	0.004	-0.93

[1]Cohen’s d
